# Is the whole greater than the sum of its parts?

**DOI:** 10.1186/1897-4287-10-S2-A45

**Published:** 2012-04-12

**Authors:** L Andrews, J Duffy

**Affiliations:** 1 Hereditary Cancer Clinic, Prince of Wales Hospital, Randwick, NSW, Australia

## 

Although mutations of BRCA2 are known to be associated with increased risks of prostate and pancreas cancer and melanoma, the prevalence of Jewish founder mutations of BRCA1 and BRCA2 (JFM’s) amongst incidental cases of these malignancies in Ashkenazi Jews has not be shown to justify founder mutation testing. This case report highlights the need to consider combinations of these cancers in Ashkenazi families.

John* (*pseudonyms), aged 63, presented to the Hereditary Cancer Clinic at Prince of Wales Hospital. All four grandparents were Ashkenazi Jewish, and he became aware of BRCA1and BRCA2 JFM testing following the identification of the BRCA2 founder mutation in the husband of his sister Judy*. John’s brother had died from pancreas cancer at 60, his father had died from prostate cancer at 69 and his uncle had died from melanoma at 60. John was known to have an elevated PSA and was being treated for benign prostatic hypertrophy. There were no cases of breast or ovarian cancer in his family.

The combination of cancers was sufficiently concerning to offer John founder mutation testing. The 6174delT mutation of BRCA2 was identified. It was also identified in his sister Judy. Despite having a husband with the same mutation, she had three adult children with no evidence of Fanconi Anaemia. John’s mother was shown not to carry the mutation, so predictive testing was offered to his paternal relatives.

To date, 14 people in this family have had predictive testing and 5 unaffected carriers have been identified. In view of his mutation result, John underwent a further prostatic biopsy and cancer was found, resulting in a prostatectomy. Two unaffected relatives have had RRSO, one was shown to have dysplastic changes in the fallopian tube. Eligible tested males have been enrolled in the IMPACT study. Mutation carriers in this family aged between 40 and 80 have been offered participation in a trial of pancreatic cancer screening with endoscopic ultrasound.

Although this family had no cases of breast or ovarian cancers, it indicates that combinations of cancers associated with BRCA2 need to be considered for testing. Despite this being a large pedigree with information about the cancer status of all members, the paternal great grandparents of the proband had 38 descendents in the next 3 generations, of whom only 11 were female, contributing to the absence of any history of breast and ovarian cancer. Families such as this highlight the need to consider population JFM testing, which is being trialled in the UK as the GCaPPS (Genetic Cancer Prediction Through Population Screening) study, and being considered for Sydney through the POWH.

